# DenTiUS Plaque, a Web-Based Application for the Quantification of Bacterial Plaque: Development and Usability Study

**DOI:** 10.2196/18570

**Published:** 2020-09-03

**Authors:** Nicolás Vila-Blanco, Vicente Freire, Carlos Balsa-Castro, Inmaculada Tomás, María J Carreira

**Affiliations:** 1 Centro Singular de Investigación en Tecnoloxías Intelixentes Universidade de Santiago de Compostela Santiago de Compostela Spain; 2 Instituto de Investigación Sanitaria de Santiago de Compostela Santiago de Compostela Spain; 3 Oral Sciences Research Group Department of Surgery and Medical Surgical Specialities, School of Medicine and Dentistry Universidade de Santiago de Compostela Santiago de Compostela Spain

**Keywords:** computer-aided diagnoses, computer-based biomedical applications, dental health, dental plaque quantification, web-based tools, medical informatics

## Abstract

**Background:**

In the dentistry field, the analysis of dental plaque is vital because it is the main etiological factor in the 2 most prevalent oral diseases: caries and periodontitis. In most of the papers published in the dental literature, the quantification of dental plaque is carried out using traditional, non-automated, and time-consuming indices. Therefore, the development of an automated plaque quantification tool would be of great value to clinicians and researchers.

**Objective:**

This study aimed to develop a web-based tool called DenTiUS and various clinical indices to evaluate dental plaque levels using image analysis techniques.

**Methods:**

The tool was executed as a web-based application to facilitate its use by researchers. Expert users are free to define experiments, including images from either a single patient (to observe an individual plaque growth pattern) or several patients (to perform a group characterization) at a particular moment or over time. A novel approach for detecting visible plaque has been developed as well as a new concept known as nonvisible plaque. This new term implies the classification of the remaining dental area into 3 subregions according to the risk of accumulating plaque in the near future. New metrics have also been created to describe visible and nonvisible plaque levels.

**Results:**

The system generates results tables of the quantitative analysis with absolute averages obtained in each image (indices about visible plaque) and relative measurements (indices about visible and nonvisible plaque) relating to the reference moment. The clinical indices that can be calculated are the following: plaque index of an area per intensity (API index, a value between 0 and 100), area growth index (growth rate of plaque per unit of time in hours; percentage area/hour), and area time index (the time in days needed to achieve a plaque area of 100% concerning the initial area at the same moment). Images and graphics can be obtained for a moment from a patient in addition to a full report presenting all the processing data. Dentistry experts evaluated the DenTiUS Plaque software through a usability test, with the best-scoring questions those related to the workflow efficiency, value of the online help, attractiveness of the user interface, and overall satisfaction.

**Conclusions:**

The DenTiUS Plaque software allows automatic, reliable, and repeatable quantification of dental plaque levels, providing information about area, intensity, and growth pattern. Dentistry experts recognized that this software is suitable for quantification of dental plaque levels. Consequently, its application in the analysis of plaque evolution patterns associated with different oral conditions, as well as to evaluate the effectiveness of various oral hygiene measures, can represent an improvement in the clinical setting and the methodological quality of research studies.

## Introduction

Dental plaque is a diverse community of microorganisms located on dental surfaces in the form of a biofilm embedded in an extracellular matrix of polymers from both the host and microbiota [1,2]. It is known that plaque is directly related to the appearance and progression of common oral pathologies like caries and periodontal diseases [3,4]. Monitoring of how it develops is, therefore, a topic of great clinical importance when it comes to establishing better strategies for the control of oral diseases caused by bacterial biofilms [5]. Several clinical indices for quantifying dental plaque have been developed over recent decades. These have been frequently used by the research community and in the clinical setting to evaluate the efficacy of different oral hygiene products. Some of these traditional plaque indices include those developed by Ramfjord [6], Greene and Vermillion [7], and Quigley and Hein [8] and later modified by Turesky et al [9], Löe [10], and O'Leary [11]. In general, most of these conventional indices employ an ordinal scale as part of a simple and semiquantitative method to evaluate surfaces covered by dental plaque. Their application, however, has major limitations, given the great subjectivity inherent in conducting visual examinations. Furthermore, the visual method is very imprecise when plaque levels are low or particularly high, and such clinical investigations are often laborious [12].

The planimetric method [13] was an improvement and involved taking a photograph of dyed plaque and determining its extent. This approach was much more accurate, as it employed a more objective measure to assess plaque levels (it produced a continuous, rather than ordinal, output). Additionally, as image sensors have improved year-on-year, the quality of the images generated is better than ever. Nevertheless, this is still a time-consuming process because the teeth and plaque regions in each image must be outlined by hand.

It was only in the 21st century that experts began to rely on techniques that employ analyses of digital images to quantify dental plaque. The main approach involved the planimetric method, with an imaging tool used to detect the dental plaque and tooth areas individually and calculate the ratio between them. An expert operator could outline both regions manually using a graphical interface [14,15]. Also possible were semiautomatic approaches, whereby the image-processing algorithm required intervention from the dental expert to work [16,17], or images could be segmented automatically using image-processing techniques [18-20].

Some researchers used image-processing software [16,17,21-23] or general-purpose data-processing tools [24,25], while others developed their own methods to process these images [24,26]. More recently, specific dental assessment software has been used to quantify plaque levels [18,27].

The computer vision techniques used previously vary from rudimentary to extremely complex. One of the simplest methods was image thresholding, which made it possible to isolate two or more different areas according to their color or light intensities. This technique was able to distinguish between disclosed plaque and nonplaque [24,28]; teeth, plaque, and gingiva pixels [23]; and isolated teeth, gums, plaque, and background areas [29,30]. More sophisticated machine learning algorithms were subsequently developed to enhance the results. Carter et al [20], for example, created a database of more than 600,000 pixels to analyze the relationship between pixel information (in both the RGB and HSI space) and pixel location (disclosed plaque, tooth, and gingivae). The information in this large table enabled a further step to be taken to create a classifier capable of labeling a pixel in a new image in its most probable location. Clustering methods have also been employed. An example is the approach adopted by Kang et al [31], who used a combination of fuzzy c-means and cellular neural network algorithms to classify image pixels into plaque, tooth surfaces, and backgrounds. A mean-shift based clustering algorithm was also used for plaque segmentation [19].

The different methods available today produce promising results, with many studies reporting the suitability of automatic or semiautomatic techniques for assessing dental plaque and, as a result, oral hygiene levels [16-18,24,27,29,32]. Specifically, quantitative light‐induced fluorescence digital (QLF-D) is an adaptation of QLF that employs a modified filter set (D007; Inspektor Research Systems BV, Amsterdam, The Netherlands), narrow‐band violet light (405 nm), and a high‐specification digital single‐lens reflex camera. This configuration has been specifically developed to enhance the visualization and quantification of dental plaque [33-35].

Following this trend of digital development [36], we present DenTiUS Plaque, which is a tool we have developed to enable the automatic assessment of dental plaque levels. DenTiUS Plaque was first envisaged as a standalone application. However, as we wanted to permit web access for expert users, a more general platform (DenTiUS Lab) was designed to separate the processing stages (DenTiUS Plaque proper module) and enable the execution of administrative tasks like user and patient management. The system also has a common interface where users can log in, include patients in the database, and interact with the DenTiUS Plaque module independently. The entire platform is web-based, so it can run on any web browser. It also has a friendly operator interface for nontechnical users.

The rest of the paper is structured as follows. The Methods section first describes the entire platform, including its main features and software architecture, and also contains a brief description of the patient database. Then, it explains DenTiUS Plaque, describing the kinds of images it can process, the experiment's design, and the processing algorithms. In the Results section, the different parameters of the quantitative analysis of dental plaque offered by the tool are presented, as well as several graphics, through a real case. Subsequently, the results of a questionnaire to individuals working in the field of dentistry on the usability of software are presented to evaluate the ease of use of the instrument and its usefulness. Finally, the paper closes with a discussion and some closing remarks and identifies possible future improvements.

## Methods

### DenTiUS Structure

**Figure 1:**
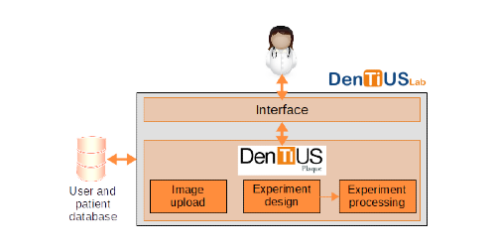
Block diagram of DenTiUS Plaque inside the DenTiUS global platform. The modular design facilitates the easy connection of the user and patient database to the DenTiUS Plaque tool.

The DenTiUS platform emerged naturally as a way to manage users, images, and patients in dentistry research. It was initially developed as a standalone application for the quantification of dental plaque, with modules for managing the patients and experiments of a unique user, but not the different profiles and interactions of multiple users. As our research group is also working in other dentistry fields, all of which employ a shared patient and user database, the decision was made to develop an entire web-based system, DenTiUS Lab, for dental assessment experiments. DenTiUS Lab integrates DenTiUS Plaque's structure into a modular platform that isolates the user interface and the patient and user database from the main DenTiUS Plaque module. Figure 1 portrays a general block diagram of the complete platform, which was designed as a content management system. This allows clinicians and researchers to sign up, register, manage patients, and interact with DenTiUS Plaque by uploading plaque images and designing and processing experiments.

DenTiUS was designed as a global platform to support clinical dental research through the inclusion of different modules that enable users to obtain a set of measures, graphs, and images with which to easily reach conclusions about their experiments. It was developed as a web application for several reasons: (1) most personal computers come with a pre-installed web browser, meaning that an installation process that could be complicated for nonspecialist users is avoided; (2) platform updates (including interface improvements, bug fixes, new features, and new modules) are deployed in a process that is completely transparent, but also unintrusive, which also ensures that all users are employing the most up-to-date application, removing any requirement to support old versions or manage compatibility issues between them; and (3) as having a web browser is the application's only requirement, it can be used on both computers and mobile devices.

**Figure 2:**
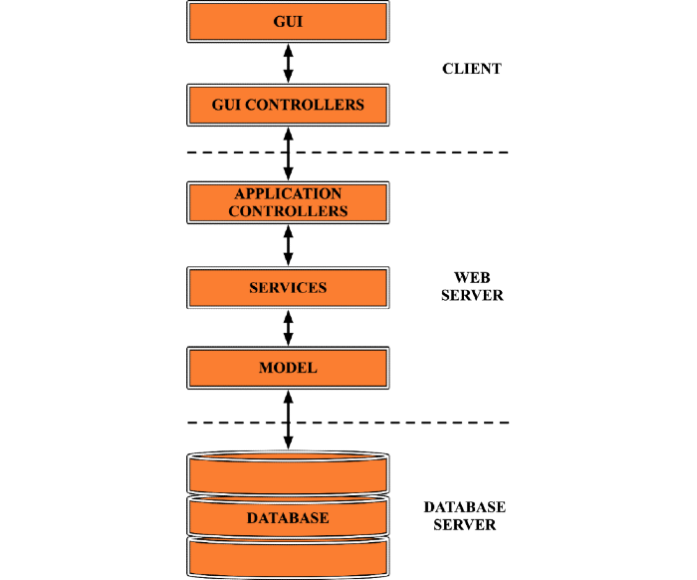
Structural diagram of the DenTiUS platform, which is divided into a client side, web server, and database server. GUI: graphical user interface.

The platform was implemented as a client-server application, which runs on a central server where all the data are stored (Figure 2). All the processing tasks are carried out on the web server, so the client does not need a high-performance device. The application logic inside the server is organized into controllers (to process user requests), services (to perform operations), and models (to manage database operations), while the well-known Model-View-Controller pattern is used to manage entities, data access, dependency injections, and many other elements of the application.

The Java programming language was employed when developing the platform because of its popularity, power, verbosity, and ease of maintenance [37]. The Model-View-Controller pattern, meanwhile, was constructed with the Spring framework [38], and data storage was managed with both Hibernate [39], which is an object-relational mapping library, and a PostgreSQL database [40]. Image processing algorithms were executed in OpenCV [41], specifically its Java implementation [42]. As dental images are usually very large, a limit of 5 Mb was set in relation to the algorithm results to ensure accuracy, with the application resizing them automatically if required.

In summary, DenTiUS was developed with maintainability and extensibility as the main objectives. In this way, the application was implemented by following a modular structure, where the visual, logic, and data layers were isolated. The functionality in each layer was divided into classes, with many abstractions available to improve the extensibility. Consequently, the only elements requiring implementation (if necessary) to develop a new module related to dental research are the data definition (eg, images, datasets), description of a new kind of experiment and its processing algorithms, and a customized report format (ie, another module with the same structure as DenTiUS Plaque, as seen in Figure 1).

**Figure 3:**
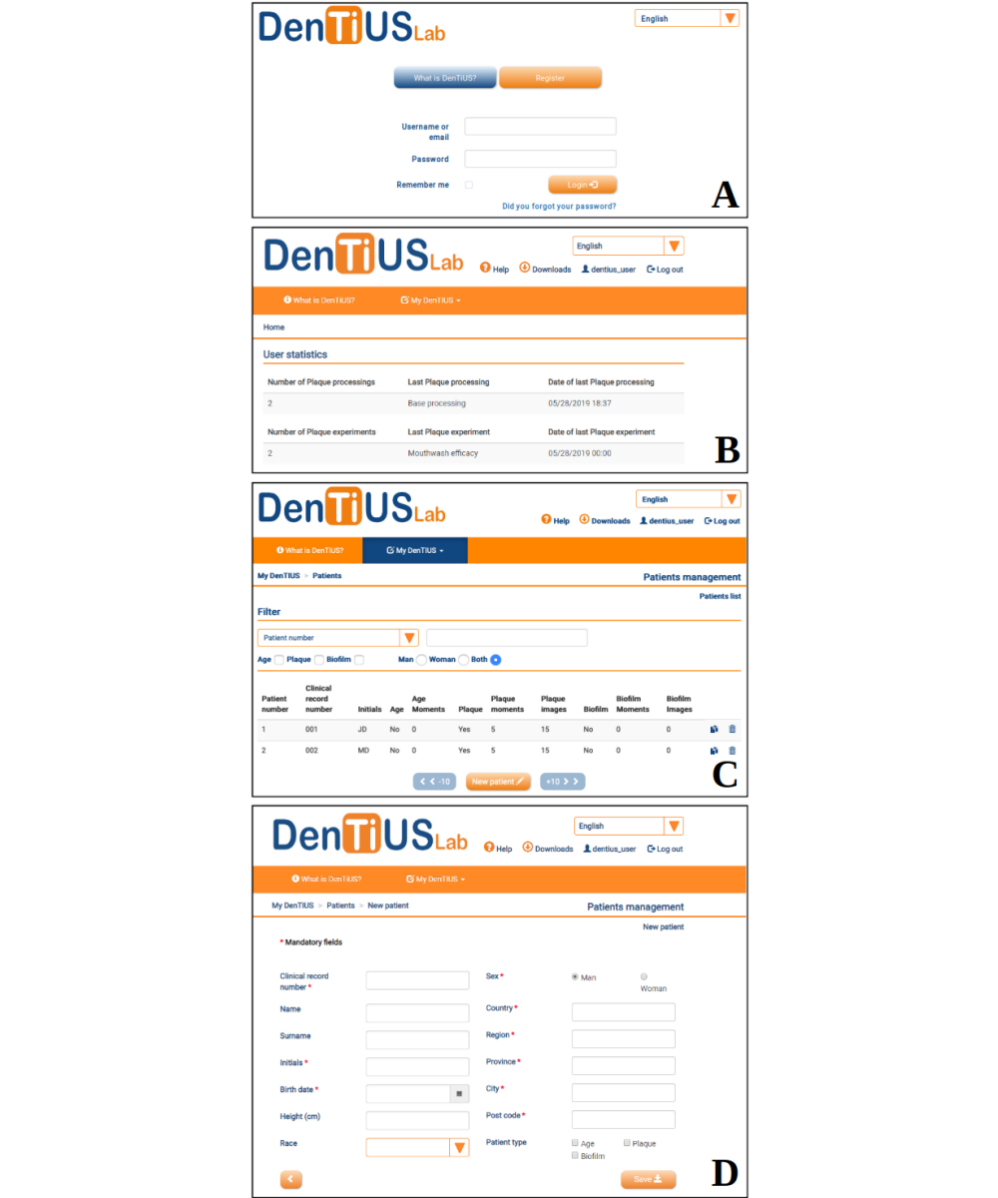
Common modules of the DenTiUS structure relating to users: (A) login screen, (B) user statistics and patients, (C) patient database, (D) new patient definition.

The DenTiUS Lab input screen (Figure 3A) asks the user to enter the application or register. If a user is new to the system, registration is a very straightforward process and takes only seconds to answer basic questions such as name, affiliation, and level of expertise. Once logged in, the system presents the statistics of use, namely finished processings, experiments, and patients (Figure 3B).

Every section of DenTiUS Lab is available from both the menu bar and side menu (visible when enough space is available). Access to other utilities is also possible through the common interface bar at the top of the screen (see Figure 3). These include a “Home” section, which is available by clicking on the DenTiUS Lab icon and is where the operator's statistics of use are displayed; “Help” page that explains the platform's features, details of the processing algorithms, and examples of use; “Downloads” section, where sample data can be downloaded for use in the application; “User” panel (accessible by clicking on the user name), where users can change their profile data and password; and “Language” section (English or Spanish). An “Admin” section is also available for users with administrative privileges.

A “Patient” database interface (Figure 3C) allows users to register and manage their patients, who can be filtered by patient number, clinical record number, patient initials, and sex. The user can also create new patient details by completing a form with data such as the patient's clinical record number, birth date, race, sex, city, and country (Figure 3D). The name and surname fields are optional to preserve patient anonymity.

### DenTiUS Plaque

#### Patient Plaque Images

DenTiUS Plaque was the main objective behind our development of the DenTiUS platform. The goal was to enable DenTiUS Plaque to perform as an automated decision support system to help experts analyze and quantify the macroscopically observable dental plaque deposited on teeth. In the first step, the software requires reproducible images of the fluorescein-dyed plaque taken under ultraviolet light. Fluorescein is a well-known fluorochrome in the field of dentistry, and a patent for its use as a dental plaque marker was filed in the USA in 1967 by Herbert Brilliant (U.S. Patent 3-309-274; 1967). In these images, the bright blue region corresponds to the tooth area unaffected by plaque, while the green region matches plaque deposits over both the tooth surfaces and gingiva.

To enable the assessment of dental plaque levels at different times, an entity called a “plaque moment” was defined for a single plaque image (usually the frontal view) or a set of images (front and lateral views) captured from a patient at a particular time. The possibility of including lateral views is a novelty of the system, as these provide a better view of the posterior teeth, where more plaque is usually accumulated.

Plaque moments can be attached to a patient's file via the Patients section (Figure 3C), which contains information about each patient concerning the number of image sets included for her or him and the number of plaque images distributed through all their moments. Another way to attach new plaque moments to a patient's record is by entering the Plaque section directly from the “MyDenTiUS” menu, where all the plaque moments are listed independently and can be modified or deleted. In any case, the user can modify, delete, or include a new moment where the images (at least one) have a date, time, brief description, and optional camera set-up parameters. At this point, it is crucial to define the so-called “reference moment” or “moment 0.” This cannot be deleted, as it is used as a reference to compute the plaque-level growth over time and usually corresponds to circumstances where there has been “perfect dental cleaning.”

#### Plaque Experiment Design

**Figure 4:**
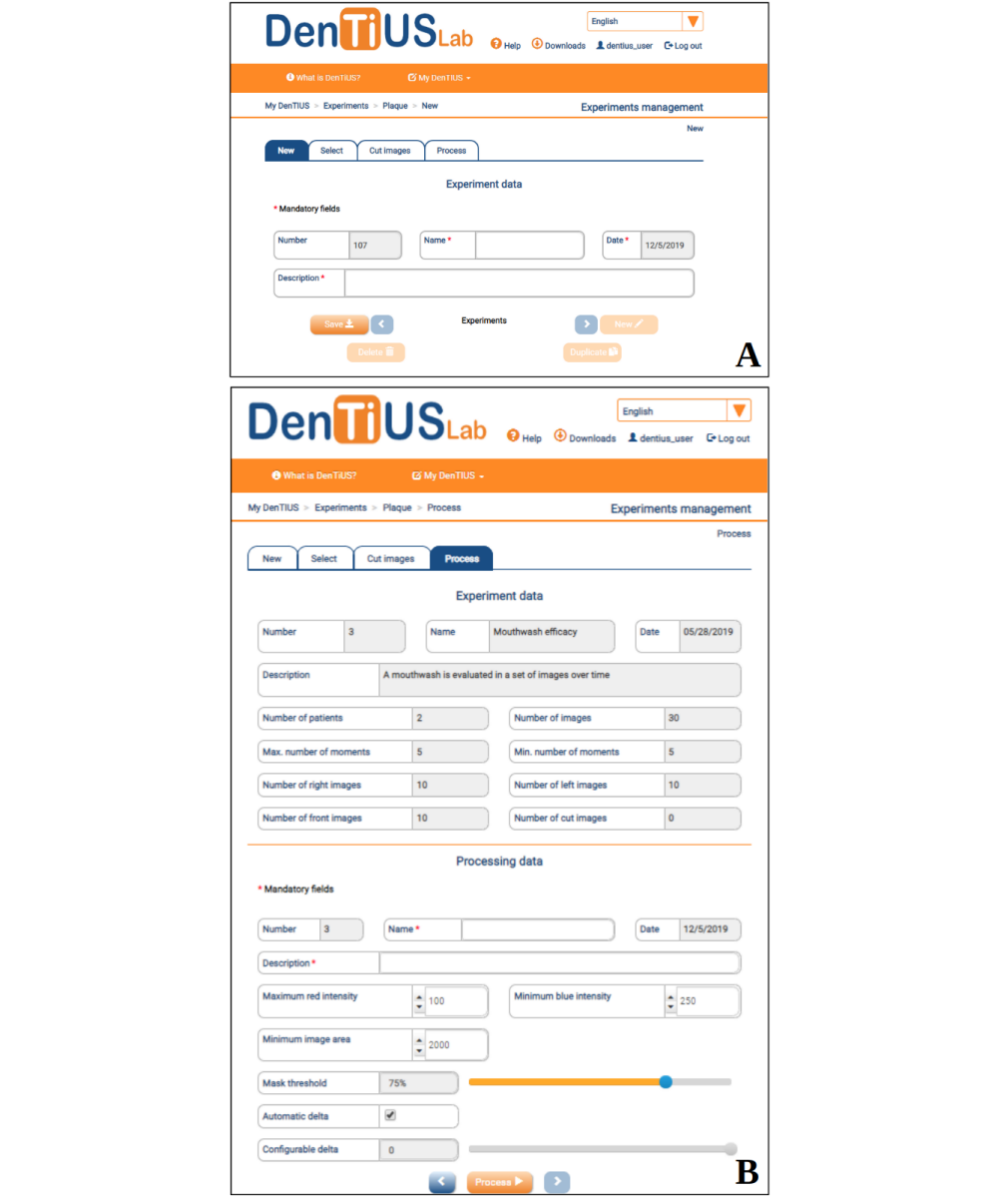
Plaque experiment design for the definition of a new experiment: (A) New tab and (B) Process tab, with a summary of the experiment and processing data showing the processing parameters that will be used when pushing the "Process" button at the bottom.

The plaque experiment design module (see Figure 1) enables users to develop new experiments by selecting specific patient data and tuning the parameters of the image processing algorithms. The experiments are presented in a list view, with several shortcuts on the right side of the screen that trigger operations like duplicating, processing, deleting, or cropping the associated images. When the user creates a new experiment, a screen appears with the following tabs: New, Select, Cut Images, and Process (Figure 4). Figure 4A shows the “New” tab, on which the name and description fields are mandatory. On the “Select” tab, the user must choose the plaque moments to be processed by selecting them from the patient database. The system automatically includes the reference moment related to any selected moment. The “Cut Images” tab allows the user to optionally select part of the images or delete artifacts. Finally, the “Process” tab (Figure 4B) shows a summary of the “Experiment data” and, below that, the “Processing data” parameters, with a mandatory name and description. This permits users to try different configurations of the processing parameters for the same set of images. In this screen, the expert user can employ the default parameters or change these to different values. The meanings of these parameters will be explained later in this section.

The configuration of the experiment is decided by the user according to the specific circumstances (age groups, gender, single or multiple patients) or objectives (eg, effectiveness of mouthwash, effectiveness of brushing devices, smoking effects). The system performs the experiments defined by the user by processing the data of all the patients and all the patient moments included in the experiment. When the experiment involves multiple moments for the same patient, the images with the selected tooth areas are subjected to a process of normalization in the number of pixels.

DenTiUS Plaque does not permit comparisons between groups, as this was not the objective of the system, but users can export all the computed parameters to manage and prepare their own statistics with no limitations.

#### Processing Algorithms

After an exhaustive analysis of the color characteristics of each region of interest, a process is defined to segment the dental area, excluding the gingiva region: Initially, the blue and red channels are added together, and the result is thresholded according to a parameter fixed by the user (by default, the algorithm chooses 75% of the darkest pixels as the background and 25% of the lightest as the foreground). The isolated pixels are then removed, and a connected component analysis is performed to identify objects from that binary image. Thereafter, a set of conditions related to region size (>2000 pixels, by default) and solidity (>0.5, by default) are applied to remove nondental regions. Other specific rules, which were defined to eliminate bright artifacts, are included to make the process fully automatic. All these parameters can be changed in the “Process” tab (see the “Processing data” parameters in Figure 4B).

Once the dental area is segmented, the system must perform an analysis of its values to extract and measure the dental plaque. As mentioned previously, plaque is characterized by a high green intensity, whereas the rest of the dental region has a high blue concentration. The first step is thus the detection of the (visible) plaque by analyzing the differences between the green and blue channels (Equation 1). The rest of the dental region is considered to be a first approximation of the nonvisible plaque area (Equation 2).

*P_visible_* = |*G-B*| for pixels with [*G-B* > 0] (**1**)

*P_nonvisible_* = |*G-B*| for pixels with [*G-B* ≤ 0 and *G*>0 and *B*>0] (**2**)

where *P_visible_* is the visible plaque, *P_nonvisible_* is the initial approximation of the nonvisible plaque, and *G* and *B* are the green and blue channels of the dental area, respectively.

**Figure 5:**
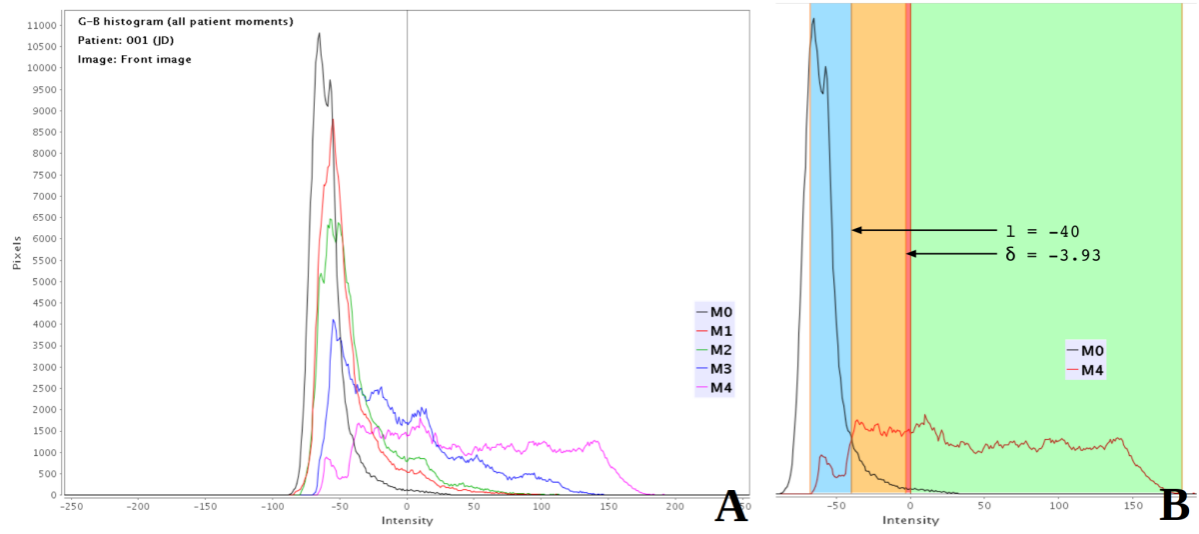
G-B histogram where the vertical line represents G-B=0 and G and B are the green and blue channels, respectively. (A) G-B histograms for moments M0 to M4, showing the progression of the histogram values toward the G-B positive values (visible plaque); (B) Definition of l and δ for M4, showing plaque (green), nonplaque (blue), level 1 nonvisible plaque (red, defined by δ), and level 2 nonvisible plaque (orange, defined by l). The boundary between nonplaque and level 2 nonvisible plaque (l) is easily observable in this chart, as it corresponds to the intensity value where the 2 histograms cross.

**Figure 6:**
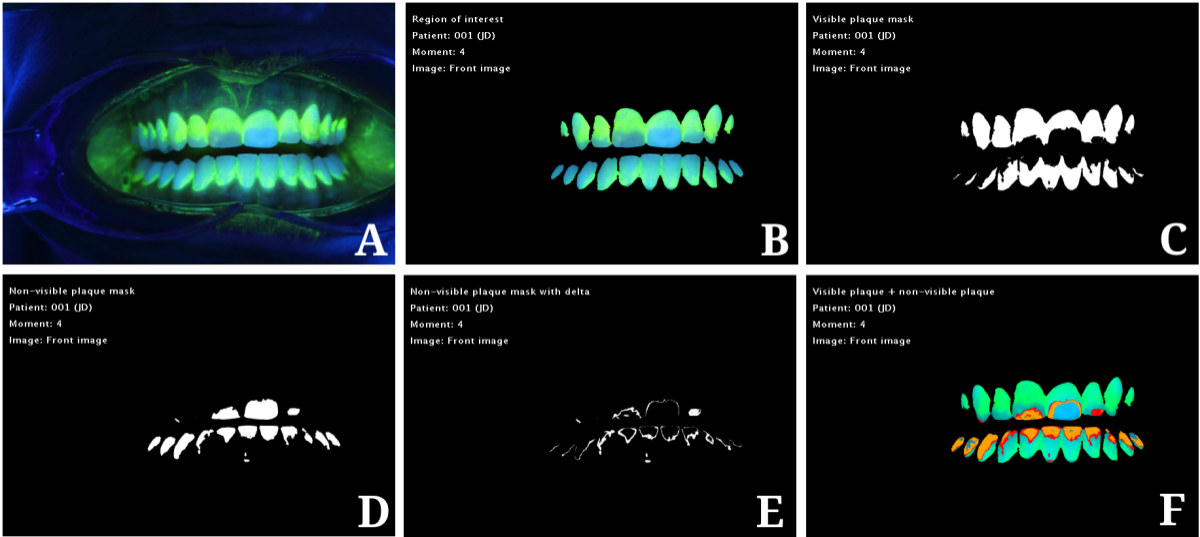
Results of the different steps of the algorithm relating to the frontal image of a patient after 96 hours of perfect cleaning: (A) frontal ultraviolet image, (B) segmented dental area, (C) visible plaque mask, (D) nonvisible plaque mask, (E) nonplaque level 1 mask, (F) final labeled image (blue: nonplaque; green: visible plaque; red: nonvisible plaque level 1; orange: nonvisible plaque level 2).

G-B dental area histograms are included to assess how levels of dental plaque increase over time when oral hygiene stops, with the positive and negative sides corresponding to the visible (G>B) and first approximation of the nonvisible plaque (G<B), respectively. Figure 5A shows an example of a 96-hour experiment, where moments were recorded every 24 hours, while Figure 6 presents the results of the different steps of the algorithm on a frontal image of a patient after 96 hours of perfect cleaning. After professional dental cleaning (moment M0), most of the histogram area corresponded to the first approximation of the nonvisible plaque area (Equation 2), as its values were highly concentrated in the negative part of the graph. In the moments that followed (M1 to M4), the histogram was flattened and displaced toward the positive side, thus increasing the visible plaque level. In the final moment (M4), 4 days after a dental cleaning, the largest part of the histogram was placed on the positive side of the graph, with the visible plaque area covering most of the dental region. The main finding behind this step was the discovery and measurement of the transition area between the visible plaque and the rest of the dental region, based on the risk of plaque developing in the near future. As a result, the system performs an extra segmentation step before displaying the nonvisible plaque area (as seen in Figure 6F).

According to 2 different parameters, *δ* and *l*, the blue/green differences are thresholded to produce 3 different region masks in the negative part of the graph, namely level 1 nonvisible plaque (risk of being plaque in the following hours), level 2 nonvisible plaque (risk of being plaque in the following days), and no plaque (no risk of being plaque in the medium term):

*P_nonvisible_(level1)* = |*G-B*| for pixels with [*G-B* ≤0 and *G-B* ≥δ] (**3**)

*P_nonvisible_(level2)* = |*G-B*| for pixels with [*G-B* <δ and *G-B* ≥l] (**4**)

*NonPlaque =* |*G-B*| for pixels with [G-B<l] (**5**)

where the *l* parameter is automatically calculated from nonvisible plaque histograms of current and reference images (Figure 5B); it is defined as the first value where the number of nonvisible plaque pixels in the current image is greater than the number of nonvisible plaque pixels in the reference image (moment 0).

The *δ* parameter can be set by the user or is automatically resolved as the absolute difference between the average pixel values of the blue and green channels in the dental area. The modification of this parameter by the user in the “Process” tab (see Figure 4B) indicates an increase or decrease in the level 1 nonvisible plaque region (red area in Figure 6F). This means that the pattern of plaque development can be simulated by starting with a minimum value and progressively increasing it.

### Ethical Approval

Images from Spanish Caucasian subjects are part of a study protocol approved by the Galician Clinical Research Ethics Committee (registration number 2014/008). The image collection was performed following the ethical standards of our institution's research committee and the 1964 Declaration of Helsinki and its later amendments [43].

## RESULTS

### Plaque Processing Results

**Figure 7:**
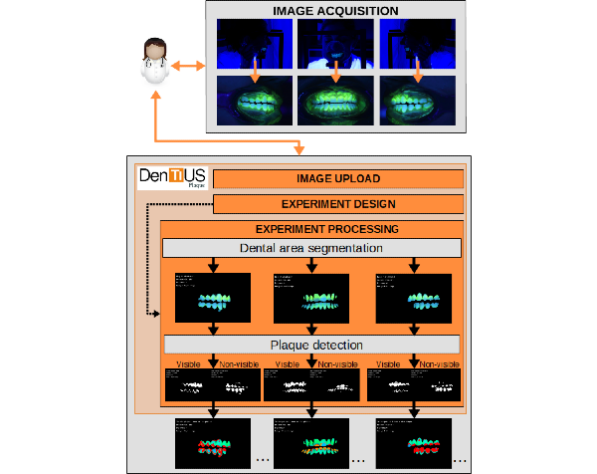
Process of detecting and quantifying dental plaque using DenTiUS Plaque for one patient moment in an experiment.

Figure 7 portrays the complete process for a single patient image set belonging to a particular experiment: Plaque images were uploaded to the database, the plaque experiment was designed, and the experiment processing was launched.

**Figure 8:**
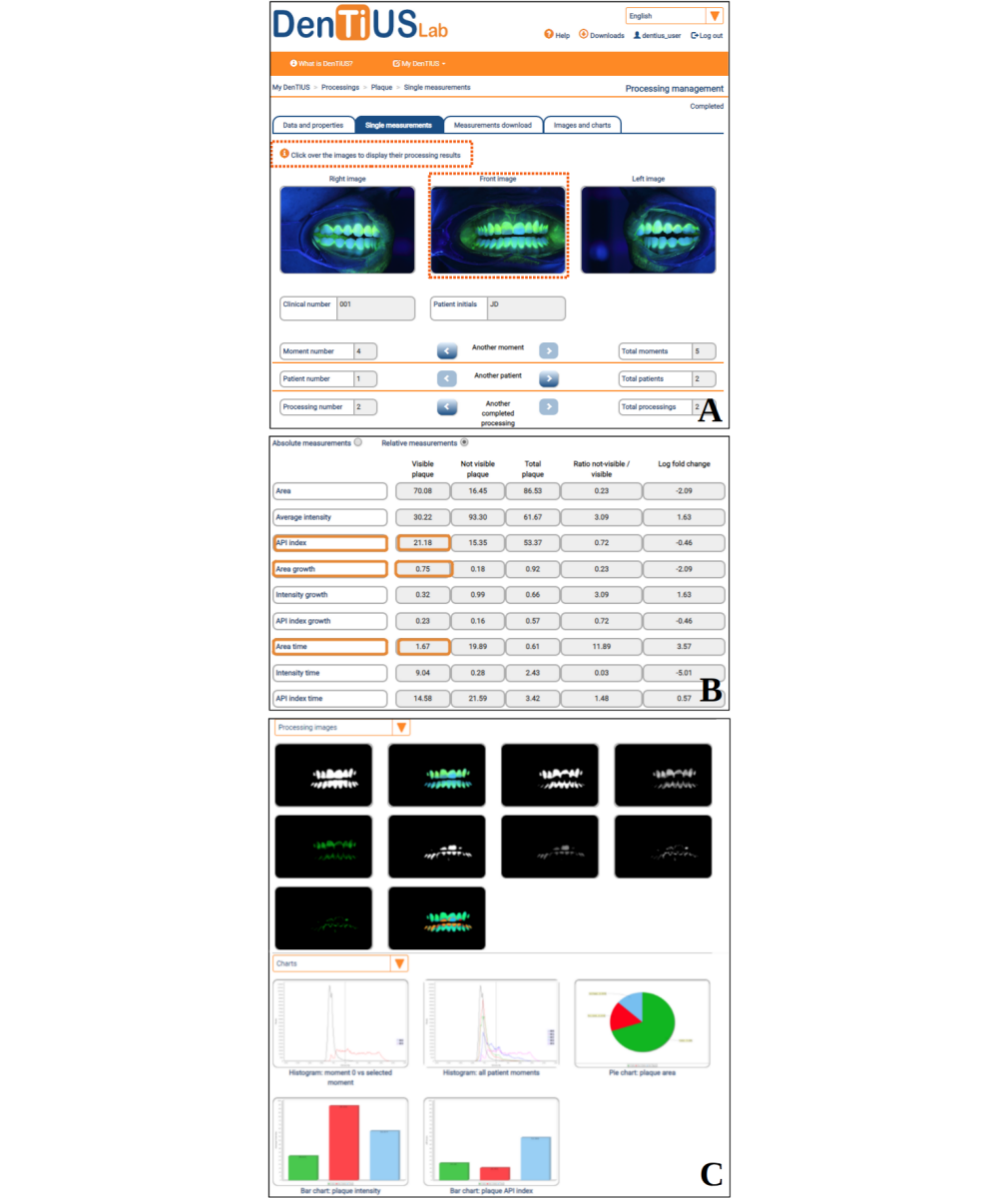
Processing results. (A) and (B) The single measurements tab showing the absolute (first column, visible plaque) and relative measurements (relative to the reference image) for the frontal image; (A) and (C) Images and charts tab for the frontal image.

When the processing finishes, the user can view the results by clicking on the appropriate notification or the “Processings” submenu. The processing results are divided into 4 tabbed subsections (Figure 8): “Data and properties,” “Single measurements,” “Measurements download,” and “Images and charts.” The “Data and properties” tab presents a summary of statistics concerning the processing as well as the processing parameters. Figure 8B shows the “Single measurements” tab, which presents the results of the quantitative analysis for each image in table form, with absolute averages obtained in each image (indices about visible plaque) and relative measurements (indices about visible and nonvisible plaque) relating to the reference moment. As shown in this figure and just below the tabs with an information alert, the user must click on each image to display their processing results in the “Single measurements” and “Images and charts” tabs.

The clinical indices defined in this research provide information on the bacterial plaque on dental surfaces, specifically the area where it is present, its intensity, a combined value between the area and intensity, and different growth parameters. The system can produce dental plaque clinical indices in a computerized form, including in relation to the plaque index of an area per intensity (API index, a value between 0-100), area growth index (growth rate of plaque per unit of time in hours: percentage area/hour), and area time index (the time, in days, needed to achieve a plaque area of 100% concerning the initial area at the same moment).

In the example in Figure 8, after 96 hours of plaque accumulation, focusing on the visible plaque values, the patient presented an API index of 21.18 and a hypothetical plaque growth rate of 0.75% per hour. A particularly interesting parameter supplying the labeled image is the area time. In this case, the area-time relative value is 1.67 for visible plaque and 0.61 for total plaque (see Figure 8B). In other words, from a theoretical point of view and assuming a constant growth pattern for that moment, the patient would take 1.67 days to achieve a visible plaque level of 100%. This value is obviously lower, around 0.61 days, when it comes to realizing plaque levels of 100% for both nonvisible and visible plaque.

The measurement tables can be customized and downloaded in CSV and spreadsheet formats via the “Measurements download” tab, where users can also select specific parameters within the absolute and relative measurements.

Figure 8C portrays an example of the images, tables, and charts obtained for one moment of one patient. The “Images and charts” tab provides not only the final view of the plaque regions in each plaque image but also the intermediate results of the computer vision algorithm, including the tooth mask, visible plaque mask, difference between the blue and green channels in both the teeth and visible plaque regions, and others. The final labeled image represents the areas with nonplaque (in blue) and visible plaque (in green). Also shown are the areas that will become plaque in the short term, graduating to near-plaque (red areas, nonvisible plaque level 1) and medium-term plaque (orange areas, nonvisible plaque level 2).

Pie and bar charts comparing the levels of visible plaque and nonvisible plaque, as well as the nonplaque areas, are also presented. Finally, 2 histograms are presented that compare the intensity distribution of the plaque in the selected image moment to the corresponding image of the reference moment and the intensity distributions of the plaque through all the moments. A vertical line at 0 can be seen in both histograms, separating values corresponding to visible plaque (Equation 1) from those relating to nonvisible plaque and nonplaque (Equations 3, 4, and 5). Finally, a full report presenting all the tables and graphs can be generated in PDF format via the main “Processings” menu. This will contain all the processing data, including all the information described previously for each processing tab.

### DenTiUS Graphical User Interface (GUI) Usability

As with most web applications, DenTiUS has a graphical user interface (GUI) that is designed to be friendly, simple, and intuitive, as well as adaptable to the various screen sizes on mobile phones, tablets, and computers. A usability test, based on the Computer System Usability Questionnaire [44], was proposed to assess the suitability of the GUI in real terms. This test was adapted to DenTiUS, resulting in 9 questions with scores ranging from 1 (strongly disagree) to 5 (strongly agree). The questions were designed to measure the following: (Q1) overall ease of use, (Q2) workflow efficiency, (Q3) user comfort, (Q4) learning process, (Q5) productivity, (Q6) value of the error messages, (Q7) online help, (Q8) attractiveness of the interface, and (Q9) overall satisfaction.

The questionnaire was sent to 34 dentists doing research in the field of dentistry, including mainly PhD researchers (28/34, 82%), undergraduate research fellows in their final year of career (3/34, 9%), and senior researchers (3/34, 9%). The testers were given instructions about how to enter DenTiUS Lab and proceed with a test case based on a patient with plaque growth over several days. In particular, they performed the following steps: signing up to the application, registering some patients and plaque moments, defining an experiment, continuing with the processing, and searching for various results.

**Figure 9:**
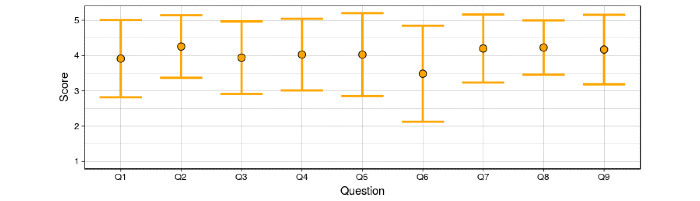
Score distribution of each usability test question. Each error bar represents the mean and standard deviation.

Figure 9 demonstrates that all the questions produced fairly good results, with a mean score of around 4 in almost all cases. The best-scoring questions were Q2, Q7, Q8, and Q9, relating to the workflow efficiency, value of the online help, attractiveness of the user interface, and overall satisfaction, respectively. By contrast, some users stated in Q6 that the platform errors should be more informative and provide better solutions to fix any issues.

### Availability of the Software

Access to the DenTiUS Lab web platform is available free of charge for non-commercial use for researchers and clinicians [45].

## Discussion

### Principal Findings

The importance of dental plaque in the etiopathogenesis of important oral diseases, such as caries and periodontitis, is well-known [5,46]; on the other hand, there are recognized limitations of conventional clinical indices of dental plaque quantification [20,23], which are widely used both in clinical and research settings. Therefore, to improve the diagnosis of dental plaque, it is essential to develop new computer systems that allow the objective quantification of dental plaque levels.

This paper introduces DenTiUS Plaque. This is a tool for the quantification of bacterial plaque integrated into a general web-based platform with a common management process for users and patients.

DenTiUS is the first dental research system to enable image collections and patient data to be managed, experiments to be designed, and images with customized configuration to be automatically processed. The developed tool produces accurate and repeatable results for the assessment of clinical indices of bacterial plaque levels relating to a patient or group of patients over time, ensuring the sustainability of the process in terms of the time and effort required by users of the system. Clinical users with no technical background can process the images in batches and obtain a table of measurements (most of which have been specifically produced for this platform) and explanatory graphs that can be exported in various formats (PDF report, spreadsheet, and JPEG images). Although a more complex final report could be designed, these different outputs were a requirement identified by dental researchers to enable them to have access to all the values needed to produce their own statistics.

Specifically, a novel algorithm was developed in DenTiUS Plaque to detect and quantify dental plaque levels from ultraviolet images. This approach first detects the dental region and then segments and quantifies visible plaque by analyzing the difference between green and blue channels. Initially, DenTiUS Plaque could represent a tool that offers the following advantages over the QFL-D system [33-35]. The first advantage is that DenTiUS Plaque allows the detection of different areas in the remaining dental regions according to the risk of developing plaque in the future; this finding was referred to as “nonvisible plaque.” DenTiUS Plaque provides the detection and quantification of nonvisible plaque at 2 levels: the probability of becoming visible plaque in the short term (level 1) or medium term (level 2). The second advantage is that DenTiUS Plaque not only provides the same indices of plaque quantification (the API index) as the QFL-D system but also indices to measure the plaque growth pattern over time for a given patient such as the area growth index and area time index. In addition, all the clinical indices developed for DenTiUS Plaque are applicable to quantifying both visible and nonvisible plaque levels.

Regarding the usability test conducted with people working in the dental field, overall, the users stated that they were likely to use the application in the future, with the results revealing that DenTiUS Plaque was suitable for its ultimate purpose.

Biological coherence between conventional clinical indices and new indices derived from image analysis is a study to be carried out to test the validity of the latter [24,47,48]. In this sense, our research group used an in situ 5-day bacterial plaque growth model to conduct some initial experiments on the validity of the DenTiUS Plaque clinical indices, comparing the results with those obtained with a conventional clinical index; to obtain both types of indices, the plaque was stained with fluorescein and displayed using ultraviolet light [49,50]. Concerning the degree of correlation between the conventional and API indices, days 1, 2, and 3 of plaque formation revealed very high correlations between the two approaches (Spearman rho ≥0.770). However, in the days where there was little or excessive accumulation of bacterial plaque, days 0 and 4, respectively, the relationship between the 2 measurement systems was suboptimal (Spearman rho ≤0.540), highlighting the limitations of the conventional index and the convenience of applying the API index produced by DenTiUS Plaque for these clinical situations. An interesting objective of future research would be to apply both DenTiUS Plaque and the QFL-D system on the same group of patients to quantify the levels of dental plaque, verifying the correlation between both systems.

### Limitations and Future Work

Further work will be conducted to improve the platform. Additional help will be included in the software based on the results of a GUI validation exercise.

The image cropping system will be improved to automatically exclude unwanted artifacts in the image. More research tools will be fully integrated into the DenTiUS platform. An example is DenTiUS Biofilm, which will enable the quantification of microscopic dental plaque as well as comparisons of the plaque growth pattern at the microscopic (biofilm) and macroscopic levels (plaque), taking advantage of the shared management of patients and users.

### Conclusions

The DenTiUS Plaque software allows automatic, reliable, and repeatable quantification of dental plaque levels, providing information about area, intensity, and growth pattern. Dentistry experts recognized that this software is suitable for quantification of dental plaque levels. Consequently, its application in the analysis of plaque evolution patterns associated with different oral conditions as well as the evaluation of the effectiveness of various oral hygiene measures can represent an improvement in the clinical setting and methodological quality of research studies.

## Abbreviations

API: area per intensity
GUI: graphical user interface
QLF-D: quantitative light‐induced fluorescence digital
